# Peripheral Arterial Disease: Erythrocyte Sedimentation Rate and Interleukin-6 as Markers of Disease Severity and Chronic Limb-Threatening Ischemia

**DOI:** 10.3390/life16101739

**Published:** 2026-10-16

**Authors:** Horatiu Comsa, Bogdan Dutu, Adina David Comsa, Gabriel Cismaru, Radu Rosu, Gabriel Gusetu, Raluca Tomoaia, Bogdan Caloian, Florina Fringu, Diana Irimie, Dana Pop

**Affiliations:** 1 4th Department of Internal Medicine, Cardiology Rehabilitation, Iuliu Hatieganu University of Medicine and Pharmacy, 400347 Cluj-Napoca, Romania; dh.comsa@gmail.com (H.C.); david.adinamaria@gmail.com (A.D.C.); rosu.radu1053@gmail.com (R.R.); florina.fringu@yahoo.com (F.F.); gurzaudiana@yahoo.com (D.I.); pop67dana@gmail.com (D.P.); 2 Cardiology Department, Clinical Rehabilitation Hospital, 400066 Cluj-Napoca, Romania; 3 Ares Cardomed, 400015 Cluj-Napoca, Romania

**Keywords:** peripheral arterial disease, inflammation, biomarkers, interleukin-6, erythrocyte sedimentation rate, chronic limb-threatening ischemia, arteriography

## Abstract

Peripheral arterial disease (PAD) is a manifestation of systemic atherosclerosis in which chronic inflammation contributes to disease severity. This retrospective study included patients who underwent arteriography for PAD at a single center in 2019. Of 238 arteriographies performed, inflammatory and hematologic biomarker panels were available for a subset of 71 patients with angiographically confirmed PAD. Serum tumor necrosis factor-alpha (TNF-α), interleukin-6 (IL-6), erythrocyte sedimentation rate (ESR), white blood cell count, platelet count, and uric acid were compared across sex, age, diabetes, and obesity subgroups, correlated with disease severity by the Leriche–Fontaine classification, and analyzed for their association with chronic limb-threatening ischemia (CLTI; Fontaine stages III–IV vs. IIA–IIB). ESR (r = 0.408, *p* = 0.001) and IL-6 (r = 0.310, *p* = 0.009) correlated significantly with disease severity, whereas TNF-α, white blood cell count, platelet count, and uric acid did not. Both markers showed modest but significant discriminative performance for CLTI (IL-6: area under the curve = 0.674, *p* = 0.012; ESR: area under the curve = 0.673, *p* = 0.021). ESR and IL-6 were both associated with PAD severity suggesting a more pronounced systemic inflammatory phenotype in CLTI; their prognostic value after revascularization warrants evaluation in larger, prospective cohorts.

## 1. Introduction

Peripheral arterial disease (PAD) is a common manifestation of systemic atherosclerosis and is associated with substantial functional impairment, cardiovascular morbidity, and mortality. Although PAD primarily affects the lower extremities, it reflects a broader atherosclerotic burden and is associated with an increased risk of major cardiovascular events [1,2].

Atherosclerosis is increasingly recognized as a chronic inflammatory disease in which endothelial dysfunction, recruitment of inflammatory cells, cytokine activation, and vascular remodeling contribute to plaque development and progression. In PAD, systemic inflammation may be particularly relevant because the disease is associated not only with atherosclerotic obstruction but also with endothelial dysfunction, impaired vasodilation, thrombosis, and reduced tissue perfusion. Several studies have demonstrated increased circulating inflammatory markers in patients with PAD and have reported associations between systemic inflammation, ABI, functional impairment, and the extent of peripheral atherosclerosis. In particular, higher concentrations of C-reactive protein (CRP), fibrinogen, and other inflammatory mediators have been associated with lower ABI values and greater clinical manifestations of peripheral arterial disease, including impaired lower-extremity functional performance; in a cohort of 370 patients with PAD and 231 without, CRP was independently associated with poorer 6-min walk distance and summary performance score among participants with PAD, but not among those without PAD [3,4,5,6].

Among circulating inflammatory mediators, interleukin-6 (IL-6) has attracted considerable interest because of its central role in the acute-phase response and its interaction with endothelial and vascular smooth-muscle cells. Increased IL-6 concentrations have been reported in patients with PAD, and previous studies have linked IL-6 and other inflammatory mediators with functional limitation and progression of peripheral atherosclerotic disease. Circulating IL-6 has been associated with functional decline in patients with lower-extremity PAD, while earlier studies demonstrated increased levels of IL-6 and tumor necrosis factor-alpha (TNF-α) in patients with PAD [7,8,9,10].

Erythrocyte sedimentation rate (ESR) represents a more traditional and readily available marker of systemic inflammation. ESR has been associated with atherosclerotic disease and has been investigated as a marker of PAD. Studies comparing patients with and without PAD have reported higher ESR values in patients with peripheral arterial disease and inverse associations between inflammatory markers, including ESR, and ABI [11]. However, in the largest prospective study to examine this relationship (the Edinburgh Artery Study), the association between IL-6 and incident PAD was substantially attenuated after adjustment for conventional cardiovascular risk factors, suggesting IL-6 may not be an independent predictor [12].

The distinction between intermittent claudication and chronic limb-threatening ischemia (CLTI) is established clinically and confirmed by hemodynamic and imaging assessment [13,14,15], and circulating biomarkers are not required for this diagnosis. A different question, however, is whether CLTI represents not only more extensive arterial obstruction but also a more pronounced systemic inflammatory phenotype. This question is relevant because inflammation has become a therapeutic target in atherosclerosis [16,17], and because markers that track the clinical severity of established PAD are logical candidates for subsequent prognostic evaluation. We therefore evaluated the association of circulating inflammatory and hematologic biomarkers with clinical disease severity according to the Leriche–Fontaine classification, compared their levels across relevant subgroups (sex, age, diabetes, and obesity), and quantified, using receiver operating characteristic analysis, the strength of their association with CLTI—as a descriptive measure of the inflammatory gradient rather than as a proposed diagnostic test.

## 2. Materials and Methods

### 2.1. Study Design and Population

This retrospective analysis included patients who underwent diagnostic and/or therapeutic peripheral arteriography for peripheral arterial disease (PAD) at the Interventional Cardiology Laboratory of the Cluj-Napoca Rehabilitation Hospital, a high-volume center, between 1 January and 31 December 2019. Procedures were performed by two independent operators, each with more than fifteen years of experience in peripheral vascular interventions. During the study period, 238 peripheral arteriographies were performed. The inflammatory cytokine panel (TNF-α and IL-6) is not part of routine care and, because of its considerable cost, was determined within a research protocol in a subset of 71 patients sampled between March and June 2019, independently of clinical stage or routine laboratory results. Seventy-one patients with angiographically confirmed PAD and available cytokine measurements (TNF-α and IL-6) formed the study population.

Inclusion criteria were age over 18 years and either disabling intermittent claudication despite optimal medical treatment or chronic limb-threatening ischemia. Exclusion criteria were acute limb ischemia, active infection, known malignancy, systemic inflammatory or autoimmune disease, and surgery or trauma within the previous 6 weeks. After arteriography, the treatment strategy was decided according to lesion characteristics, clinical status, and operator judgment: percutaneous transluminal angioplasty (PTA; plain balloon angioplasty, stenting, or both), surgical revascularization, a combined approach, or conservative treatment.

### 2.2. Clinical and Laboratory Data Collection

Demographic data (age, sex, residence), cardiovascular risk factors (smoking status, hypertension, dyslipidemia, diabetes mellitus, obesity), clinical PAD stage according to the Fontaine classification, ankle–brachial index (ABI), and type of treatment (percutaneous revascularization, surgical revascularization, combined, or conservative) were extracted from patient records. Venous blood samples were collected at the time of the index procedure for determination of serum inflammatory and hematologic biomarkers, including high-sensitivity C-reactive protein (hs-CRP), tumor necrosis factor-alpha (TNF-α), interleukin-6 (IL-6) using ELISA method (BioVendor a.s., Brno, Czech Republic), erythrocyte sedimentation rate (ESR), white blood cell (WBC) count, platelet count, and serum uric acid.

### 2.3. Statistical Analysis

Continuous variables were tested for normality using the Shapiro–Wilk test. Normally distributed variables are presented as mean ± standard deviation (SD) and were compared between groups using the independent-samples *t*-test; non-normally distributed variables are presented as median (interquartile range, IQR) and were compared using the Mann–Whitney U test. Categorical variables are presented as absolute and relative frequencies. Associations between biomarker levels and PAD severity (Fontaine stage, treated as an ordinal variable) were assessed using Spearman’s rank correlation coefficient (r).

The association of individual biomarkers with chronic limb-threatening ischemia (CLTI) was quantified using receiver operating characteristic (ROC) curve analysis. For the purpose of this retrospective analysis, CLTI was operationally defined as Fontaine stages III–IV (ischemic rest pain, ulceration, or gangrene) versus stages IIA–IIB, consistent with the clinical presentations included in the CLTI definition of the Global Vascular Guidelines [14]. Although widely used for its simplicity, Fontaine stage IV encompasses a broad spectrum of tissue loss, from small ischemic ulcers to extensive gangrene, which is characterized more precisely by the Society for Vascular Surgery WIfI (Wound, Ischemia, and foot Infection) classification, grading wound extent and depth, gangrene, ischemia, and foot infection [18] as well as by the Rutherford classification, which distinguishes minor (category 5) from major (category 6) tissue loss [19]. 

The area under the curve (AUC) and corresponding 95% confidence intervals were calculated for each biomarker. Optimal cut-off values were selected by maximizing the Youden index (sensitivity + specificity − 1); because they were derived and evaluated in the same sample, they are likely to overestimate discriminative performance in other cohorts. The 95% confidence intervals of the AUC were obtained by non-parametric bootstrap with 2000 resamples. All analyses were performed on available cases for each biomarker, without imputation. The number of patients with available data for each biomarker is reported below. The associations of ESR and IL-6 levels with the presence of CLTI were further examined using multivariable logistic regression models adjusted for age, diabetes, and smoking status. The relationship between biomarkers and ABI was assessed by Spearman correlation in patients with available ABI values, excluding non-compressible arteries (ABI > 1.40). A two-tailed *p*-value < 0.05 was considered statistically significant throughout. Statistical analyses were performed using SPSS version 23.

### 2.4. Ethical Approval

The study was conducted in accordance with the Declaration of Helsinki and was approved by the Institutional Ethics Committee (No. 6082/2019).

## 3. Results

Prior to analysis, hs-CRP values were excluded because inter-patient variability was implausibly low for an inflammatory marker (coefficient of variation of 2%, range 10,204–11,349 ng/mL across the entire cohort), suggesting a systematic pre-analytical or data-recording error rather than genuine biological variation; this parameter should be re-examined at the source-data level before being reconsidered in future analyses.

### 3.1. Baseline Characteristics

Seventy-one patients with peripheral arterial disease (PAD) were included in the study (Table 1). The mean age was 65.8 ± 9.6 years, and the cohort was predominantly male (59.2%) and lived in urban areas (63.4%). Cardiovascular risk factors were common: 64.8% of patients were smokers, 73.2% had hypertension, 57.7% had dyslipidemia, 39.4% had diabetes mellitus, and 16.9% were obese. Regarding disease severity, most patients presented with Fontaine stage IIB (49.3%), followed by stage III (23.9%) and stage IV (23.9%), and 2.8% presented with stage IIA. Combining stages III and IV, 34 patients (47.9%) met the definition of chronic limb-threatening ischemia (CLTI). After arteriography, 45.1% of patients were treated by percutaneous revascularization, 29.6% by surgical revascularization, and 5.6% by a combined approach, whereas 18.3% were managed conservatively. Among the total cohort, balloon angioplasty (28.2%) was more frequent than stenting (21.1%).

### 3.2. Biomarker Levels Across Clinical Subgroups

Comparisons of inflammatory and hematologic biomarkers (TNF-α, IL-6, ESR, white blood cell [WBC] count, platelet count, and uric acid) across clinically relevant subgroups are presented in Table 2. Serum uric acid was significantly higher in male than in female patients (6.46 [5.81–7.37] vs. 5.70 [4.68–7.02] mg/dL, *p* = 0.037). WBC count was significantly higher in patients younger than 65 years compared with those aged 65 years or older (9.57 ± 2.75 vs. 7.85 ± 2.32 ×10^9^/L, *p* = 0.009). Platelet count showed a non-significant trend toward higher values in women (*p* = 0.058). No other biomarker differed significantly between sexes or age groups, and no significant differences were observed between diabetic and non-diabetic patients, or between obese and non-obese patients, for any biomarker assessed—although the obese subgroup was small (*n* = 12) and the corresponding comparisons may have been underpowered.

### 3.3. Association Between Biomarkers and Disease Severity

**Figure 1:**
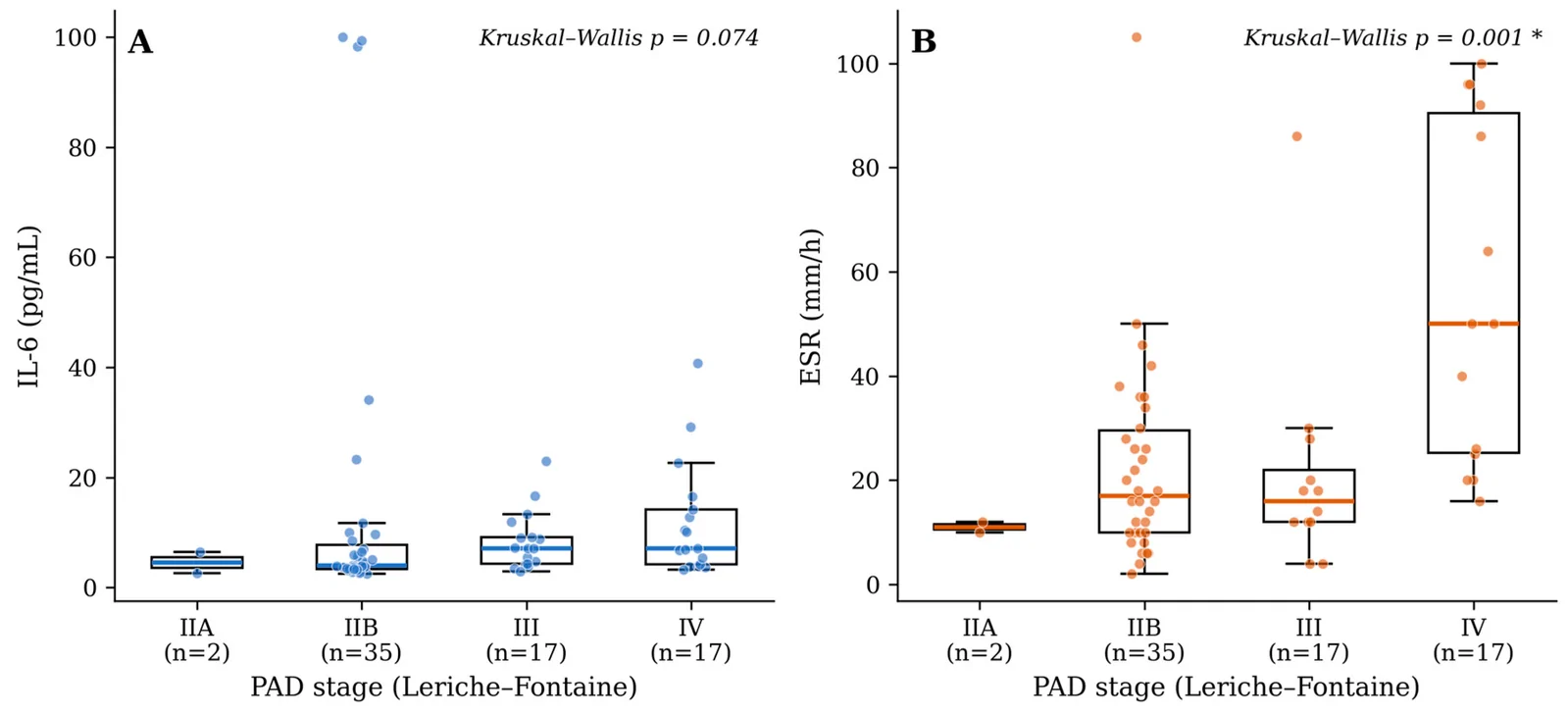
Serum IL-6 (**A**) and ESR (**B**) levels across Leriche–Fontaine PAD stages (IIA–IV). Boxplots show median and interquartile range; individual patient values are overlaid. *p*-Values from the Kruskal–Wallis test across the four stages. *: *p* value < 0.05.

Spearman correlation analysis showed that ESR and IL-6 were significantly associated with PAD stage, coded ordinally from IIA to IV (ESR: r = 0.408, *p* = 0.001; IL-6: r = 0.310, *p* = 0.009) (Table 3). Both markers increased progressively across stages, with the most pronounced gradient observed for ESR (Figure 1). TNF-α, WBC count, platelet count, and uric acid did not correlate significantly with disease stage.

**Figure 2:**
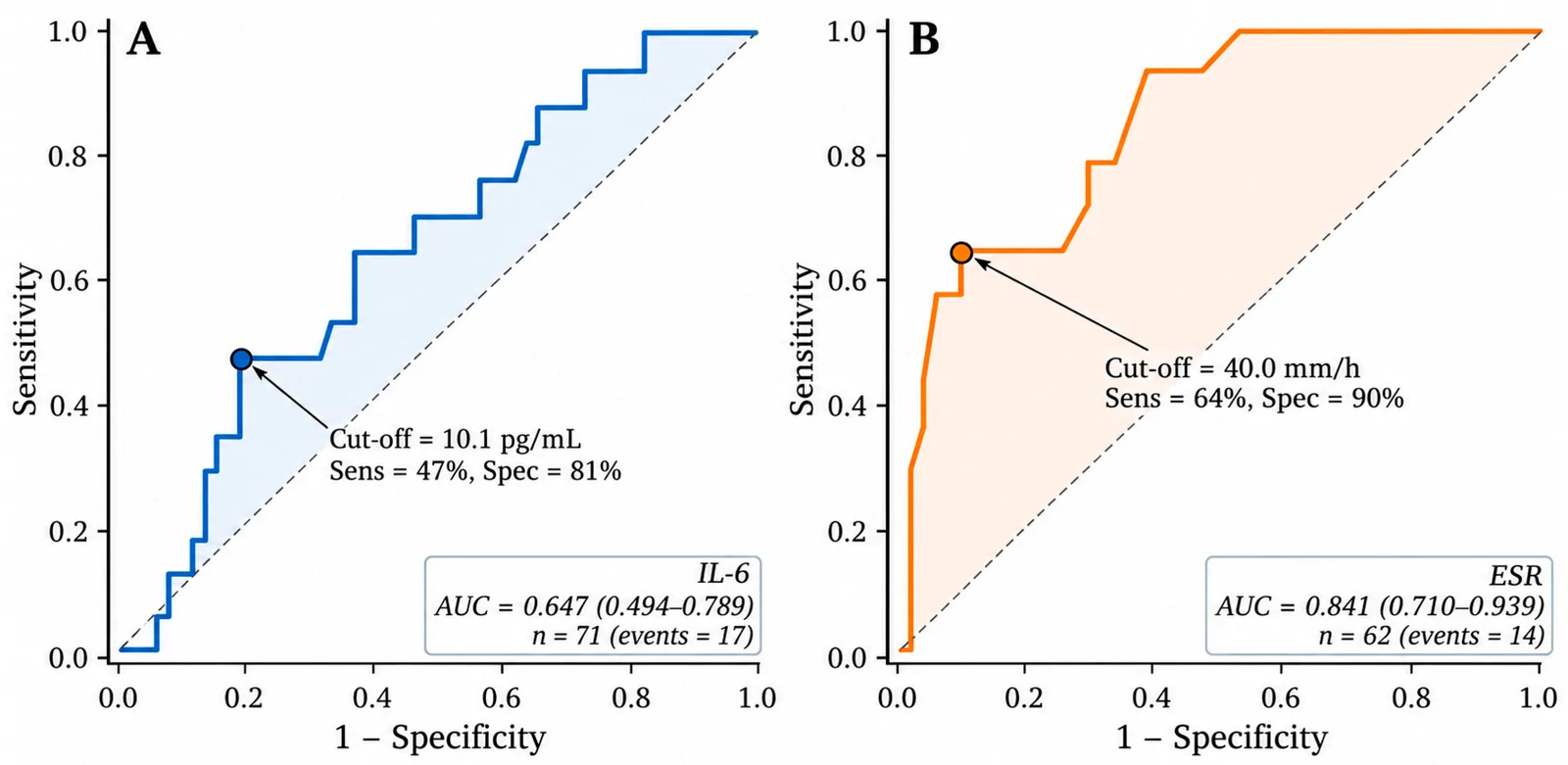
Receiver operating characteristic (ROC) curves for IL-6 (**A**) and ESR (**B**) for chronic limb-threatening ischemia (CLTI; Fontaine stages III–IV vs. IIA–IIB). The marked point indicates the optimal (Youden index) cut-off with corresponding sensitivity and specificity. AUC: area under the curve (95% confidence interval).

Given this association, the strength of the association of ESR and IL-6 with chronic limb-threatening ischemia (CLTI; Fontaine stages III–IV, *n* = 34) versus less severe disease (stages IIA–IIB, *n* = 37) was evaluated by receiver operating characteristic (ROC) analysis (Table 3, Figure 2). IL-6 showed modest but significant discriminative ability, with an area under the curve (AUC) of 0.674 (95% CI: 0.538–0.798, *p* = 0.012). The optimal cut-off was 6.7 pg/mL, corresponding to a sensitivity of 62% and a specificity of 73%. ESR showed comparable overall performance (AUC = 0.673, 95% CI: 0.515–0.803, *p* = 0.021), but with a markedly different sensitivity/specificity profile: at the optimal cut-off of 50.0 mm/h, sensitivity was 35% and specificity was 94%. Thus, markedly elevated ESR values were largely confined to patients with CLTI, whereas higher IL-6 values were distributed across a larger proportion of these patients. Given the moderate number of events (*n* = 34), these cut-off estimates should be regarded as exploratory pending external validation.

### 3.4. Inter-Marker Correlations

**Figure 3:**
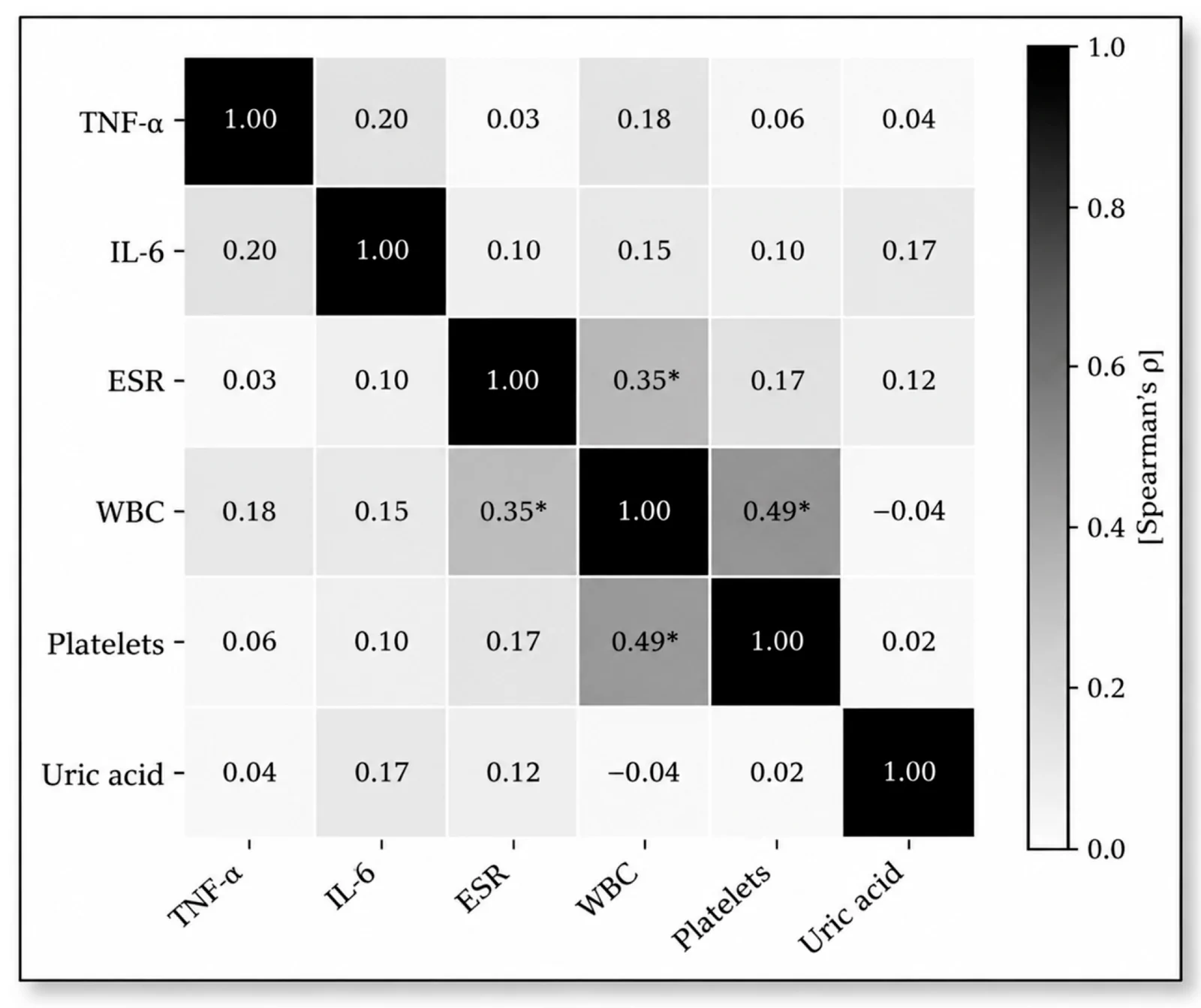
Spearman correlation matrix among inflammatory and hematologic biomarkers (TNF-α, IL-6, ESR, WBC count, platelet count, uric acid). *: *p* value < 0.05.

To assess whether the studied biomarkers provide overlapping or complementary information, pairwise Spearman correlations were computed among all inflammatory and hematologic parameters (Figure 3). Overall, correlations were weak-to-moderate, indicating that most markers behaved largely independently of one another. The strongest associations were observed between WBC count and platelet count (r = 0.49, *p* < 0.05) and between WBC count and ESR (r = 0.35, *p* < 0.05), consistent with their shared role in the acute inflammatory/hematologic response. Notably, ESR and IL-6— the two markers associated with disease severity—were only weakly correlated with each other (r = 0.10), suggesting that they capture distinct, complementary aspects of the inflammatory process underlying PAD severity.

## 4. Discussion

### 4.1. Principal Findings

This study evaluated a panel of inflammatory and hematologic biomarkers in 71 patients with angiographically confirmed peripheral arterial disease (PAD) undergoing peripheral arteriography. Erythrocyte sedimentation rate (ESR) and interleukin-6 (IL-6) were significantly associated with disease severity according to the Leriche–Fontaine classification, whereas tumor necrosis factor-alpha (TNF-α), white blood cell (WBC) count, platelet count, and uric acid were not. When the strength of their association with chronic limb-threatening ischemia (CLTI; Fontaine stages III–IV) was quantified by ROC analysis, IL-6 and ESR showed comparable, modest discriminative performance (AUC = 0.674 and 0.673, respectively), with markedly different sensitivity/specificity profiles. Subgroup comparisons by sex, age, diabetes, and obesity revealed only two significant differences (higher uric acid in men and higher WBC count in younger patients).

### 4.2. Inflammation and Disease Severity in Peripheral Arterial Disease

Our finding that ESR and IL-6, but not TNF-α or hematologic cell counts, tracked Fontaine stage is consistent with the broader literature indicating that systemic inflammatory activation in PAD is graded rather than uniform and tends to concentrate in specific acute-phase pathways rather than affecting all circulating markers equally. This is consistent with a wider conceptual framework in which atherosclerosis is understood as a chronic inflammatory disease of the vessel wall, from plaque initiation through progression and rupture [20,21]. In the InCHIANTI population-based study, IL-6, IL-1 receptor antagonist, fibrinogen, and C-reactive protein were all significantly higher in community-dwelling adults with PAD (ankle–brachial index [ABI] < 0.90) than in those without, although these associations were attenuated after adjustment for physical activity level [4]. Ünlü et al. similarly reported that inflammatory and hemostatic markers were significantly higher in patients with PAD and showed a significant inverse relationship with the ABI, reinforcing the concept that circulating inflammatory burden scales with hemodynamic severity rather than merely marking disease presence [5]. More recently, Ceasovschih et al., in a cohort of 296 participants, confirmed that ESR, fibrinogen, C-reactive protein, and other markers were significantly higher in PAD and inversely correlated with both the ABI and claudication distance [11]. Our results extend this pattern specifically to the severity gradient within an already-diagnosed PAD population rather than to the presence/absence of PAD. In our cohort, ESR and IL-6 were not significantly correlated with ABI. The interpretation of ABI is, however, limited in advanced PAD by the high prevalence of non-compressible tibial arteries, particularly in diabetic patients and in those with tissue loss. Moreover, ABI was available in 62 patients; therefore, the incremental value of ESR and IL-6 beyond ABI could not be reliably assessed and should be addressed in future studies using toe pressures or transcutaneous oximetry.

### 4.3. IL-6 as a Marker of Peripheral Arterial Disease Severity

IL-6 correlated significantly with Fontaine stage (r = 0.310, *p* = 0.009) and showed modest but significant diagnostic performance for chronic limb-threatening ischemia (CLTI; AUC = 0.674, *p* = 0.012). A similar severity-related trend was reported by Danielsson et al., who found IL-6 significantly elevated specifically in patients with critical limb ischemia, but not in those with less advanced intermittent claudication, when comparing across Fontaine stages [22]. Signorelli et al. also reported significantly higher resting IL-6 (alongside TNF-α) in PAD patients compared with healthy controls [10], supporting a real, graded elevation of IL-6 across the PAD spectrum. In contrast, an earlier and considerably smaller study by Fiotti et al.—eight patients with intermittent claudication, eight with critical limb ischemia, and eight controls—found no significant association between IL-6 and the presence of PAD, although IL-1 receptor antagonist was highest among patients with critical limb ischemia [23]. This discrepancy most likely reflects the very small sample size of that early study rather than a genuine absence of effect and underscores the value of adequately powered cohorts, including the present one, for detecting graded, severity-dependent associations. In the Edinburgh Artery Study (1519 subjects, 17-year follow-up), IL-6 was significantly associated with incident PAD only in analyses adjusted for age and sex; once conventional cardiovascular risk factors were accounted for, the association lost statistical significance [12]. In the present cohort, however, IL-6 retained statistical significance for CLTI even at conventional thresholds, suggesting that its discriminative value may be more evident when the outcome captures the full spectrum of limb-threatening disease (stages III–IV) rather than the single most severe stage alone.

### 4.4. ESR as an Accessible Marker of Advanced PAD

ESR showed the strongest correlation with disease stage (r = 0.408, *p* = 0.001) among all markers assessed and retained statistically significant, though more modest, discriminative performance for chronic limb-threatening ischemia (CLTI; AUC = 0.673, 95% CI: 0.515–0.803, *p* = 0.021) than when CLTI was restricted to Fontaine stage IV alone (AUC = 0.841 in a post hoc comparison, indicating that the marked rise in ESR is most pronounced in the most advanced stage of disease rather than across the full CLTI spectrum. This AUC is comparable to the value reported by Ceasovschih et al. for ESR in discriminating PAD presence from non-PAD controls (AUC = 0.682) [11]. ESR was not among the markers evaluated in the Edinburgh Artery Study, precluding a direct prospective comparison [10]; however, in that cohort, C-reactive protein, fibrinogen, and haematocrit—markers physiologically related to the same acute-phase and rheological pathway as ESR—retained independent significance even after full adjustment for cardiovascular risk factors. Notably, ESR values above 50 mm/h were observed almost exclusively in patients with CLTI (specificity 94%), although only about one-third of these patients exceeded this threshold (sensitivity 35%).

### 4.5. TNF-α: Absence of a Significant Association

TNF-α did not correlate significantly with Fontaine stage (r = 0.124, *p* = 0.302) nor discriminate chronic limb-threatening ischemia (CLTI; AUC = 0.595, *p* = 0.288) in our cohort. This null finding should be interpreted cautiously, as it is only partly consistent with the prior literature. Danielsson et al. reported a limited acute TNF-α response to angioplasty itself, with elevation detected in only 1 of 10 patients after balloon dilation [24], and, in a separate comparison across Fontaine stages, found TNF-α significantly elevated specifically in critical limb ischemia [22]—only partly compatible with our null result, even though the CLTI definition used here (stages III–IV, *n* = 34) provided considerably more statistical power than a stage IV-only comparison. In contrast, Signorelli et al. reported significantly higher resting TNF-α levels in PAD patients than in controls, with higher TNF-α associated with lower ABI (i.e., greater severity) [10], which is not consistent with our findings. Possible explanations for this discrepancy include differences in sampling timing relative to the ischemic or interventional insult, assay sensitivity and calibration range, and population differences.

### 4.6. Relationship Between Inflammatory and Hematologic Biomarkers

Beyond TNF-α, WBC count, platelet count, and uric acid were also not significantly associated with PAD severity in our cohort. However, WBC count correlated significantly with both platelet count (r = 0.49) and ESR (r = 0.35), consistent with their shared origin in the acute inflammatory/hematologic response, while showing only weak correlation with IL-6 and TNF-α. This pattern suggests that the cytokine-driven (IL-6, TNF-α) and cellular (WBC, platelets) arms of the inflammatory response, while biologically linked, do not necessarily move in parallel within individual patients and that each may capture a partially distinct facet of the underlying process. Among subgroup analyses, WBC count was significantly higher in patients younger than 65 years than in older patients (9.57 ± 2.75 vs. 7.85 ± 2.32 ×10^9^/L, *p* = 0.009); this somewhat counterintuitive finding may reflect a more robust acute-phase leukocyte response in younger patients, or a lower burden of chronic comorbidity-related leukocyte suppression, though this interpretation remains speculative given the cross-sectional design. Uric acid was significantly higher in men than in women (6.46 vs. 5.70 mg/dL, *p* = 0.037), consistent with well-established sex differences in renal urate handling; dietary intake and renal function, both plausible confounders, were not available for adjustment in this dataset. Neither diabetes nor obesity was significantly associated with any biomarker, but the corresponding subgroups were small (28 diabetic and 12 obese patients), and these analyses were likely underpowered to detect modest effects. Beyond the specific markers assessed here, CRP has also been linked to postoperative outcomes after lower-extremity vein bypass surgery [25] and to functional impairment among community-dwelling older adults with PAD [26], while elevated levels of a broader panel of circulating inflammatory markers have been documented in PAD populations more generally [27]. These findings reinforce the view that systemic inflammation in PAD extends well beyond the specific markers assessed in the present cohort.

### 4.7. Correlations Between Biomarkers

Pairwise correlations among the six biomarkers studied were generally weak-to-moderate, indicating that most markers behaved largely independently of one another. This relative independence argues against redundancy and supports the use of a multi-marker panel rather than reliance on any single biomarker, since each may capture a partially distinct aspect of the inflammatory or hemostatic response. This rationale parallels the approach taken by Ceasovschih et al., who combined neopterin, fibrinogen, and cystatin C—markers with only moderate mutual correlation—into a single multivariable logistic model to improve diagnostic performance for PAD rather than relying on any one marker alone [11].

Peripheral arterial disease affects a substantial proportion of the general population, and its burden is expected to grow further with population aging and the persistence of major atherosclerotic risk factors [1,2]. Most prior evaluations of inflammatory markers in PAD derive from considerably larger cohorts than the present study: 1519 subjects with 17 years of follow-up in the Edinburgh Artery Study [12], 955 participants in InCHIANTI [4], and 296 participants in the cohort reported by Ceasovschih et al. [11], compared with the 71 patients evaluated here. Our findings should therefore be interpreted as hypothesis-generating within this broader context rather than as confirmatory, particularly for the CLTI analyses (*n* = 34 events). To our knowledge, this is among the first studies to evaluate this specific inflammatory and hematologic biomarker panel—with an explicit focus on severity stratification and the association with CLTI—in an Eastern European, Romanian PAD population undergoing peripheral angiography.

### 4.8. Clinical Implications

Our findings do not support the use of ESR or IL-6 for the diagnosis of CLTI, which remains a clinical diagnosis based on symptoms, hemodynamic measurements, and imaging [13,14,15]. Rather, they indicate that patients with CLTI carry a higher systemic inflammatory burden than patients with disabling claudication and that this gradient is reflected by two largely independent markers (r = 0.10 between ESR and IL-6). The ROC-derived thresholds should be interpreted as descriptive of the magnitude of this association in our cohort. The clinical relevance of this inflammatory phenotype—in particular, whether ESR or IL-6 measured before revascularization predicts limb outcomes and whether patients with the highest inflammatory burden could benefit from anti-inflammatory strategies [16,17]—remains to be established in prospective outcome studies.

This interpretation should also consider the marked clinical heterogeneity of CLTI. In the multicenter CLIMATE Italian Registry, which included 2399 patients with CLTI from 36 vascular centers, age over 75 years, nephropathy, coronary artery disease, and the presence of dry or wet gangrene/infection were independent predictors of 1-year mortality [28]. Several of these determinants—renal dysfunction, tissue loss, and infection—also influence circulating inflammatory markers. Inflammatory biomarkers should therefore be interpreted alongside established clinical and systemic risk factors rather than in isolation, and future prognostic studies should evaluate whether ESR or IL-6 add information beyond these factors.

### 4.9. Non-Atherosclerotic Determinants of ESR and IL-6

ESR and IL-6 are non-specific markers of inflammation, and several factors other than the severity of atherosclerotic disease may have contributed to the associations observed. First, Fontaine stage IV includes ischemic ulcers and gangrene, frequently accompanied by local infection, which directly increase ESR and IL-6; the higher discriminative performance of ESR when CLTI was restricted to stage IV (AUC = 0.841) suggests that tissue loss and infection account for a substantial part of the ESR signal. Second, active smoking, present in 64.8% of our patients, is associated with higher IL-6 and acute-phase protein levels. Third, chronic kidney disease increases IL-6 through reduced clearance and increases ESR through anemia and elevated fibrinogen, and renal function was not available for adjustment. Fourth, statins and other cardiovascular drugs may lower inflammatory marker levels, and medication at the time of sampling was not systematically recorded. Finally, ESR is also influenced by age, sex, anemia, and concomitant inflammatory or neoplastic disease. Given the modest number of events, multivariable adjustment for these factors was not feasible. The associations reported here should therefore be interpreted as reflecting the overall inflammatory burden of patients with advanced PAD—including that generated by tissue loss and infection—rather than as a specific marker of atherosclerotic activity.

### 4.10. Strengths and Limitations

This study was conducted at a high-volume interventional center with two operators, each having more than fifteen years of experience, and evaluated multiple biomarkers simultaneously in a well-characterized, angiographically confirmed cohort. Its main limitations are the retrospective design, the modest sample size (*n* = 71), the single-center setting, and the absence of external validation. CLTI was defined using the Fontaine classification (stages III–IV) rather than the more granular WIfI [16] or Rutherford [17] classifications, as wound-, perfusion-, and infection-specific data were not systematically available. In particular, Fontaine stage IV groups together patients with small ischemic ulcers and those with extensive gangrene, and the use of Fontaine stages III–IV as a surrogate for CLTI may have introduced misclassification; this represents an approximation of contemporary CLTI staging rather than a full implementation of it. These limitations, particularly the single-center design and the use of a Fontaine-based CLTI approximation, warrant caution in generalizing the proposed cut-offs beyond this cohort. Because the cytokine panel was available for only 71 of the patients undergoing angiography during the study period, a selection bias cannot be fully excluded. Finally, follow-up clinical outcomes (major adverse limb events, major amputation, repeat revascularization, and mortality) were not analyzed, and the treatment strategy was heterogeneous; therefore, the prognostic value of ESR and IL-6 after revascularization could not be assessed. This represents the most clinically relevant question raised by our findings. Potential confounders of ESR and IL-6, including renal function, medication, and the presence and extent of infection, were not available for adjustment. Finally, the ROC-derived cut-offs were derived and evaluated in the same sample, without internal cross-validation or external validation, and are therefore likely to be optimistic.

### 4.11. Future Perspectives

The most important next step is to determine whether pre-procedural ESR and IL-6 predict major adverse limb events, amputation, and repeat revascularization after percutaneous or surgical revascularization, in a prospective cohort with standardized follow-up. In addition, prospective, multicenter studies with larger cohorts are needed to externally validate the ESR and IL-6 cut-offs proposed here and to confirm their association with disease severity. Future work should also evaluate whether combining ESR and IL-6, or extending the biomarker panel to include markers such as neopterin or cystatin C [9], improves discrimination of chronic limb-threatening ischemia (CLTI) beyond either marker alone. β2-Microglobulin, previously proposed as a biomarker of vascular inflammation and arterial stiffness in PAD, represents another candidate for such an extended panel [29].

## 5. Conclusions

In this cohort of patients with PAD undergoing peripheral arteriography, ESR and IL-6 were both significantly associated with disease severity and were higher in patients with chronic limb-threatening ischemia (CLTI), indicating a more pronounced systemic inflammatory phenotype in advanced disease. These markers are not proposed as diagnostic tests for CLTI, which remains a clinical diagnosis. Given the modest sample size, these findings are hypothesis-generating and require external validation; the prognostic value of ESR and IL-6 after revascularization should be evaluated in larger, prospective cohorts with clinical follow-up.

## Figures and Tables

**Table 1:** Baseline characteristics of the study population (N = 71).

Characteristic	Value
Age, years (mean ± SD)	65.8 ± 9.6 (*n* = 70)
Male sex, *n* (%)	42 (59.2)
Female sex, *n* (%)	29 (40.8)
Urban residence, *n* (%)	45 (63.4)
Rural residence, *n* (%)	26 (36.6)
Smokers, *n* (%)	46 (64.8)
Hypertension, *n* (%)	52 (73.2)
Dyslipidemia, *n* (%)	41 (57.7)
Diabetes mellitus, *n* (%)	28 (39.4)
Obesity, *n* (%)	12 (16.9)
Overweight, *n* (%)	1 (1.4)
**PAD stage (Leriche–Fontaine)**	
Stage IIA, *n* (%)	2 (2.8)
Stage IIB, *n* (%)	35 (49.3)
Stage III, *n* (%)	17 (23.9)
Stage IV, *n* (%)	17 (23.9)
**Type of treatment**	
Percutaneous revascularization (PTA), *n* (%)	32 (45.1)
Surgical revascularization, *n* (%)	21 (29.6)
Conservative (no revascularization), *n* (%)	13 (18.3)
Combined percutaneous and surgical, *n* (%)	4 (5.6)
**PTA type**	
Balloon angioplasty, *n* (%)	20 (28.2)
Stent, *n* (%)	15 (21.1)

**Table 2:** Comparison of inflammatory and hematologic biomarkers across clinical subgroups.

**Sex**			
Biomarker	Male (*n* = 42)	Female (*n* = 29)	*p*-Value
TNF-α, pg/mL	7.91 (6.49–9.97)	8.94 (7.39–10.49)	0.159
IL-6, pg/mL	6.16 (3.69–10.33)	4.33 (3.71–7.14)	0.479
ESR, mm/h	19.00 (10.00–36.50)	20.00 (12.50–34.50)	0.705
WBC, ×10^9^/L^2^	8.41 ± 2.52	8.74 ± 2.82	0.617
Platelets, ×10^9^/L	231.00 (194.00–279.00)	257.50 (236.25–320.50)	0.058
Uric acid, mg/dL	6.46 (5.81–7.37)	5.70 (4.68–7.02)	0.037 *
**Age group**			
Biomarker	<65 years (*n* = 30)	≥65 years (*n* = 41)	*p*-value
TNF-α, pg/mL	8.68 (6.87–10.49)	8.94 (6.87–9.97)	0.766
IL-6, pg/mL	4.82 (3.64–12.92)	6.42 (3.80–9.04)	0.820
ESR, mm/h	19.00 (12.00–28.50)	20.00 (12.00–37.50)	0.675
WBC, ×10^9^/L^2^	9.57 ± 2.75	7.85 ± 2.32	0.009 *
Platelets, ×10^9^/L	263.50 (228.25–306.50)	237.00 (196.00–273.00)	0.124
Uric acid, mg/dL	6.18 (5.12–6.99)	6.32 (5.50–7.63)	0.303
**Diabetes mellitus**			
Biomarker	Diabetic (*n* = 28)	Non-diabetic (*n* = 43)	*p*-value
TNF-α, pg/mL	8.94 (7.26–10.87)	8.42 (6.36–9.97)	0.422
IL-6, pg/mL	5.20 (3.71–9.97)	5.67 (3.64–9.73)	0.897
ESR, mm/h	20.00 (16.00–37.50)	19.00 (10.00–31.50)	0.199
WBC, ×10^9^/L^2^	8.69 ± 3.21	8.45 ± 2.21	0.736
Platelets, ×10^9^/L	237.00 (218.50–271.00)	256.00 (212.25–300.25)	0.480
Uric acid, mg/dL^2^	6.50 ± 1.53	6.34 ± 1.68	0.696
**Obesity**			
Biomarker	Obese (*n* = 12)	Non-obese (*n* = 58)	*p*-value
TNF-α, pg/mL	8.42 (6.87–11.39)	8.94 (6.49–9.97)	0.749
IL-6, pg/mL	7.94 (3.96–15.60)	5.26 (3.71–9.71)	0.350
ESR, mm/h	19.00 (12.00–24.50)	20.00 (12.00–39.00)	0.711
WBC, ×10^9^/L	8.90 ± 1.98	8.42 ± 2.73	0.505
Platelets, ×10^9^/L	279.00 (215.50–314.50)	243.00 (213.00–276.00)	0.521
Uric acid, mg/dL	6.57 ± 2.04	6.40 ± 1.53	0.794

TNF-α: tumor necrosis factor-alpha; IL-6: interleukin-6; ESR: erythrocyte sedimentation rate; WBC: white blood cell count. *: *p* value < 0.05.

**Table 3:** Correlation of inflammatory and hematologic biomarkers with disease severity and their association with chronic limb-threatening ischemia.

Biomarker	Correlation with PAD Severity ^1^		ROC Analysis for Chronic Limb-Threatening Ischemia (CLTI; Stages III–IV vs. IIA–IIB) ^2^			
	Spearman’s r	*p*-Value	AUC (95% CI)	*p*-Value	Cut-Off	Sens./Spec.
TNF-α, pg/mL	0.124	0.302	0.595 (0.433–0.702)	0.288	7.91 pg/mL	71%/49%
IL-6, pg/mL	0.310	0.009 *	0.674 (0.538–0.798)	0.012 *	6.74 pg/mL	62%/73%
ESR, mm/h	0.408	0.001 *	0.673 (0.515–0.803)	0.021 *	50.0 mm/h	35%/94%
WBC, ×10^9^/L	0.216	0.075	0.588 (0.453–0.717)	0.212	8.58 × 10^9^/L	58%/64%
Platelets, ×10^9^/L	0.145	0.234	0.550 (0.412–0.686)	0.478	234 × 10^9^/L	70%/50%
Uric acid, mg/dL	0.093	0.458	0.513 (0.371–0.665)	0.867	6.22 mg/dL	60%/56%

TNF-α: tumor necrosis factor-alpha; IL-6: interleukin-6; ESR: erythrocyte sedimentation rate; WBC: white blood cell count; AUC: area under the receiver operating characteristic curve; CI: confidence interval (bootstrap, 2000 resamples); Sens.: sensitivity; Spec.: specificity. *: *p* value < 0.05. ^1^ Correlation analysis between biomarker levels and PAD severity. ^2^ ROC analysis evaluating the ability of biomarkers to discriminate between patients with CLTI and those with stages IIA–IIB. Number of patients with available data: TNF-α (71); IL-6 (71); ESR (71); WBC (71); platelets (71); uric acid (71). Cut-offs were selected by the Youden index and were derived and evaluated in the same sample.

## Data Availability

The original data presented in the study are openly available in Figshare repository Inflammation_database at: https://doi.org/10.6084/m9.figshare.33417316.

## Abbreviations

The following abbreviations are used in this manuscript:

PAD	Peripheral arterial disease
ABI	Ankle–brachial index
CLTI	Chronic limb-threatening ischemia
PTA	Percutaneous transluminal angioplasty
TNF-α	Tumor necrosis factor-alpha
IL-6	Interleukin-6
ESR	Erythrocyte sedimentation rate
WBC	White blood cell (count)
hs-CRP	High-sensitivity C-reactive protein
ROC	Receiver operating characteristic
AUC	Area under the (ROC) curve
CI	Confidence interval
SD	Standard deviation
IQR	Interquartile range
